# Supplementary figures and images for: Importance of mobile genetic elements for dissemination of antimicrobial resistance in metagenomic sewage samples across the world
Source: PLoS One. 2023 Oct 19;18(10):e0293169. doi: 10.1371/journal.pone.0293169 (PMC10586675; doi:10.1371/journal.pone.0293169)

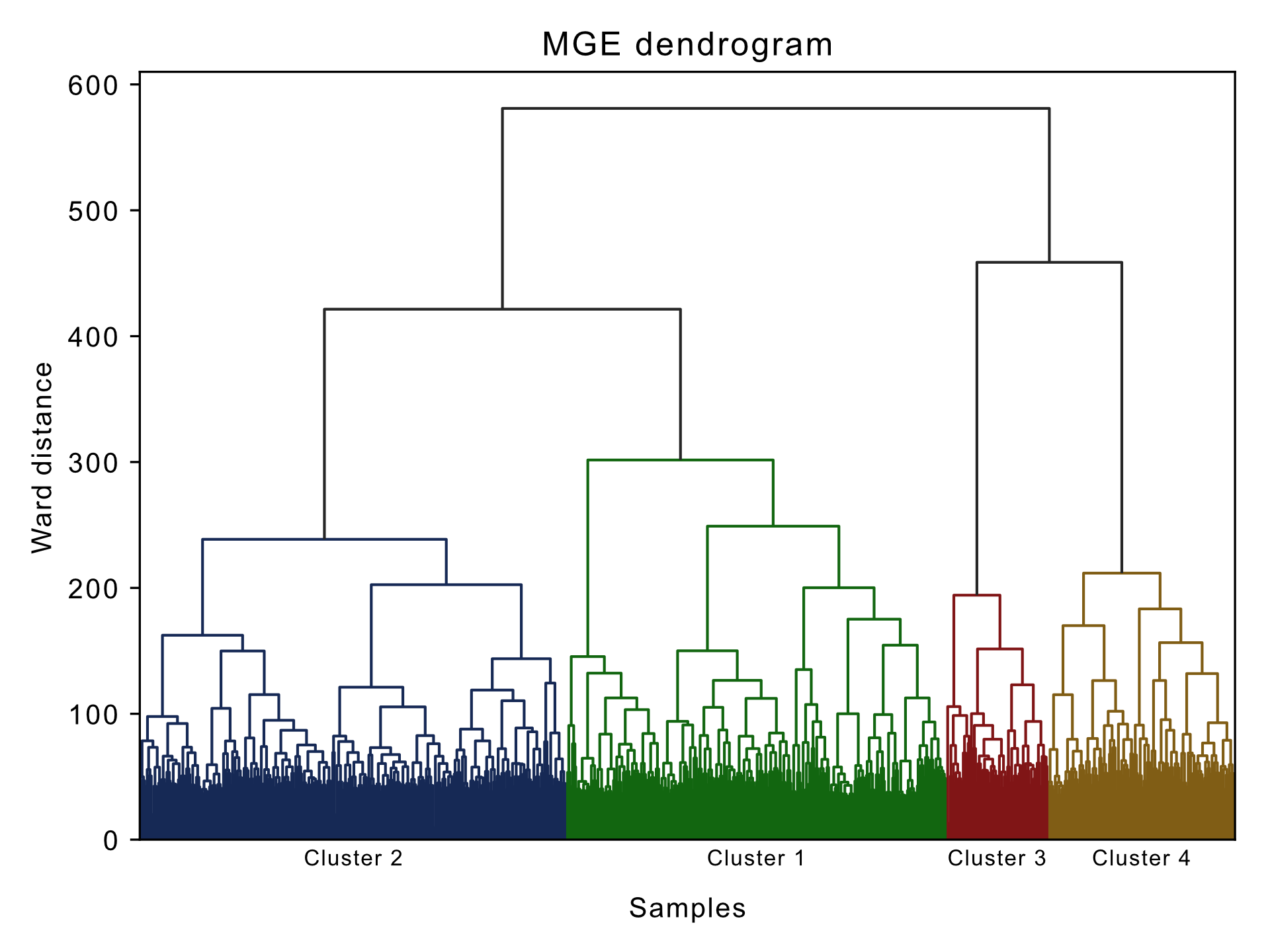

Supplement: S1 Fig — The four clusters are colored and named after the geographical clustering in Fig 2. (TIF) [file pone.0293169.s001.tif]

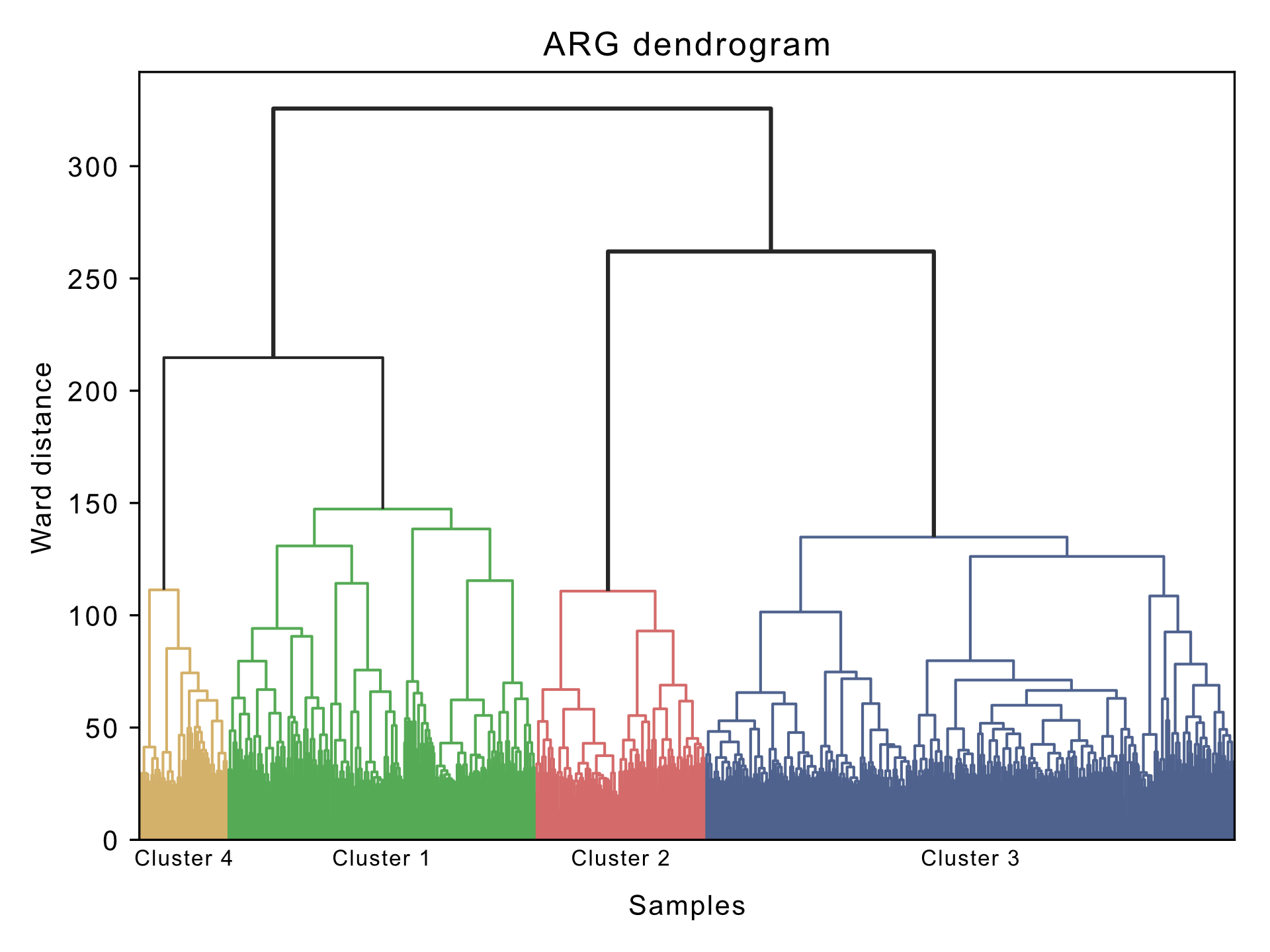

Supplement: S2 Fig — The four clusters are colored and named after the geographical clustering in Fig 2. (TIF) [file pone.0293169.s002.tif]

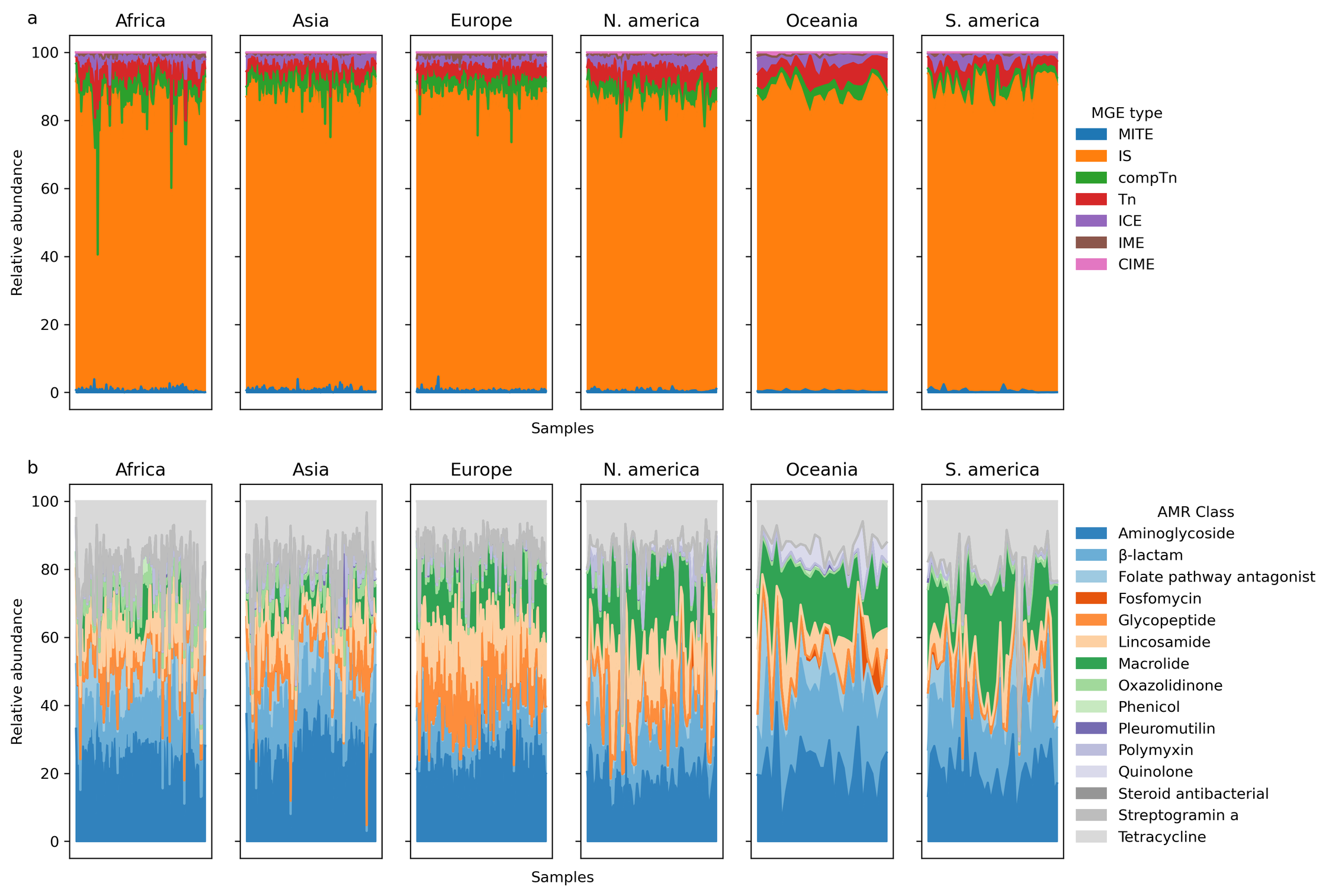

Supplement: S3 Fig — Per sample abudance of MGEs was closed to 100 to display changes in the relative abundance within and between continents. b Relative FPKM transformed abundance of ARGs per continent. ARG abundance are grouped on the antibiotic class the gene yeilds resistance to. ARGs without assiged antibiotic class was excluded. (TIF) [file pone.0293169.s003.tif]

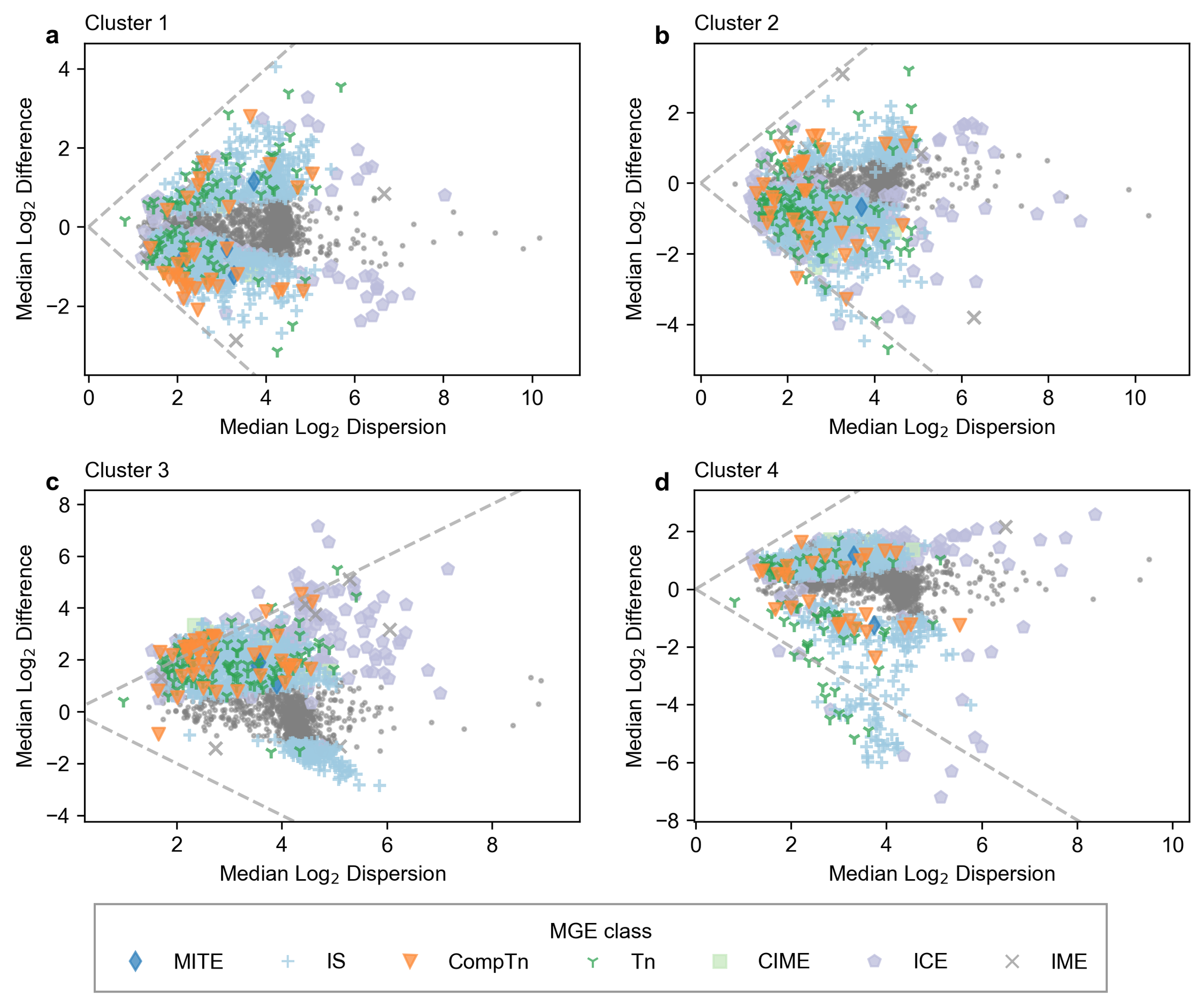

Supplement: S4 Fig — The relation of between cluster difference and within cluster dispersion of CLR transformed MGE abundances. Diagonal line show effect size of 1. MGEs with significant differential abundance (Benjamin-Hochberg corrected P value < 0.05) are colored according to MGE type. (TIF) [file pone.0293169.s004.tif]

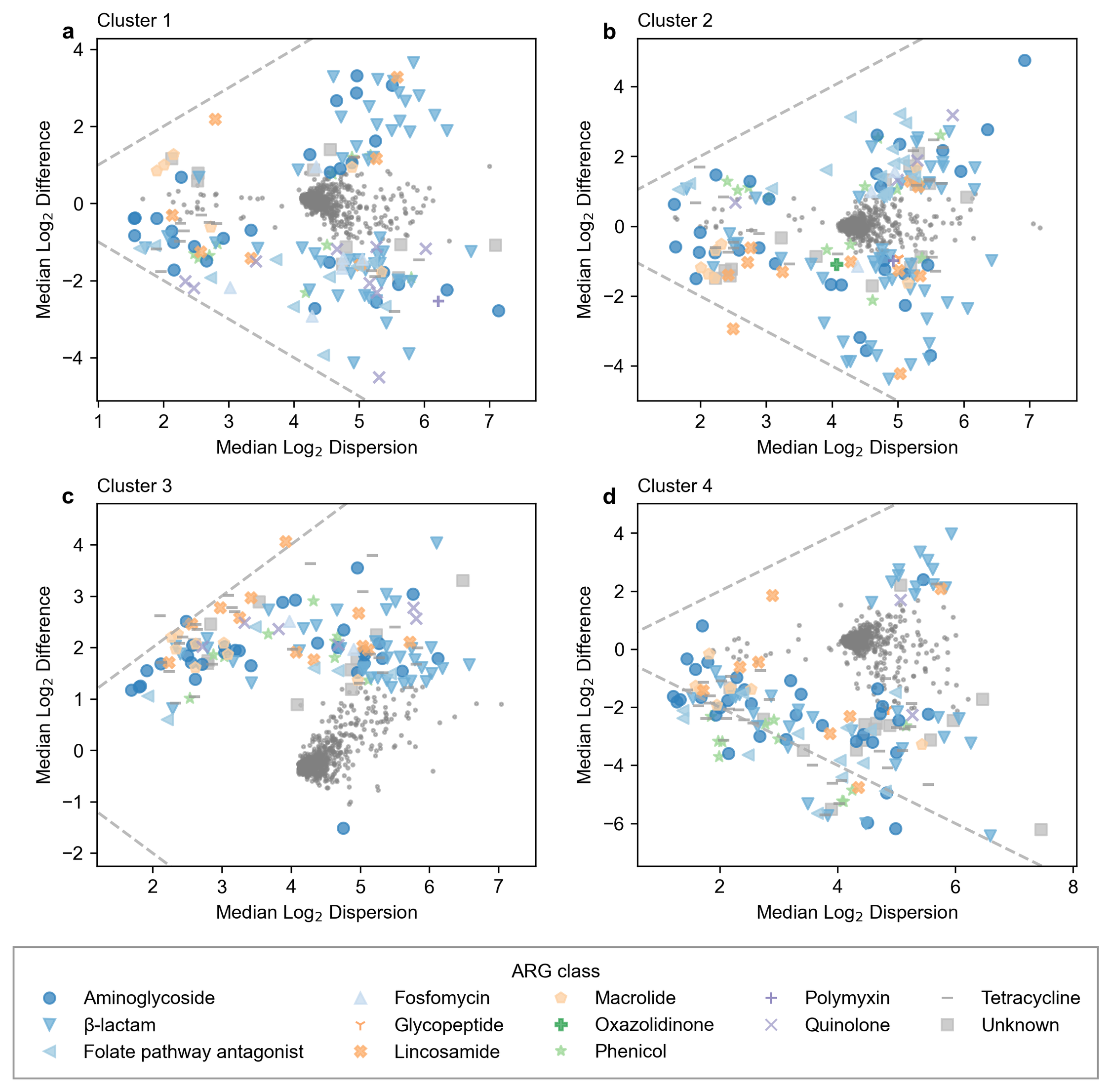

Supplement: S5 Fig — The relation of between cluster difference and within cluster dispersion of CLR transformed ARG abundances. Diagonal line show effect size of 1. MGEs with significant differential abundance (Benjamin-Hochberg corrected P value < 0.05) are colored according to the antibiotic the gene yield resistance to. (TIF) [file pone.0293169.s005.tif]

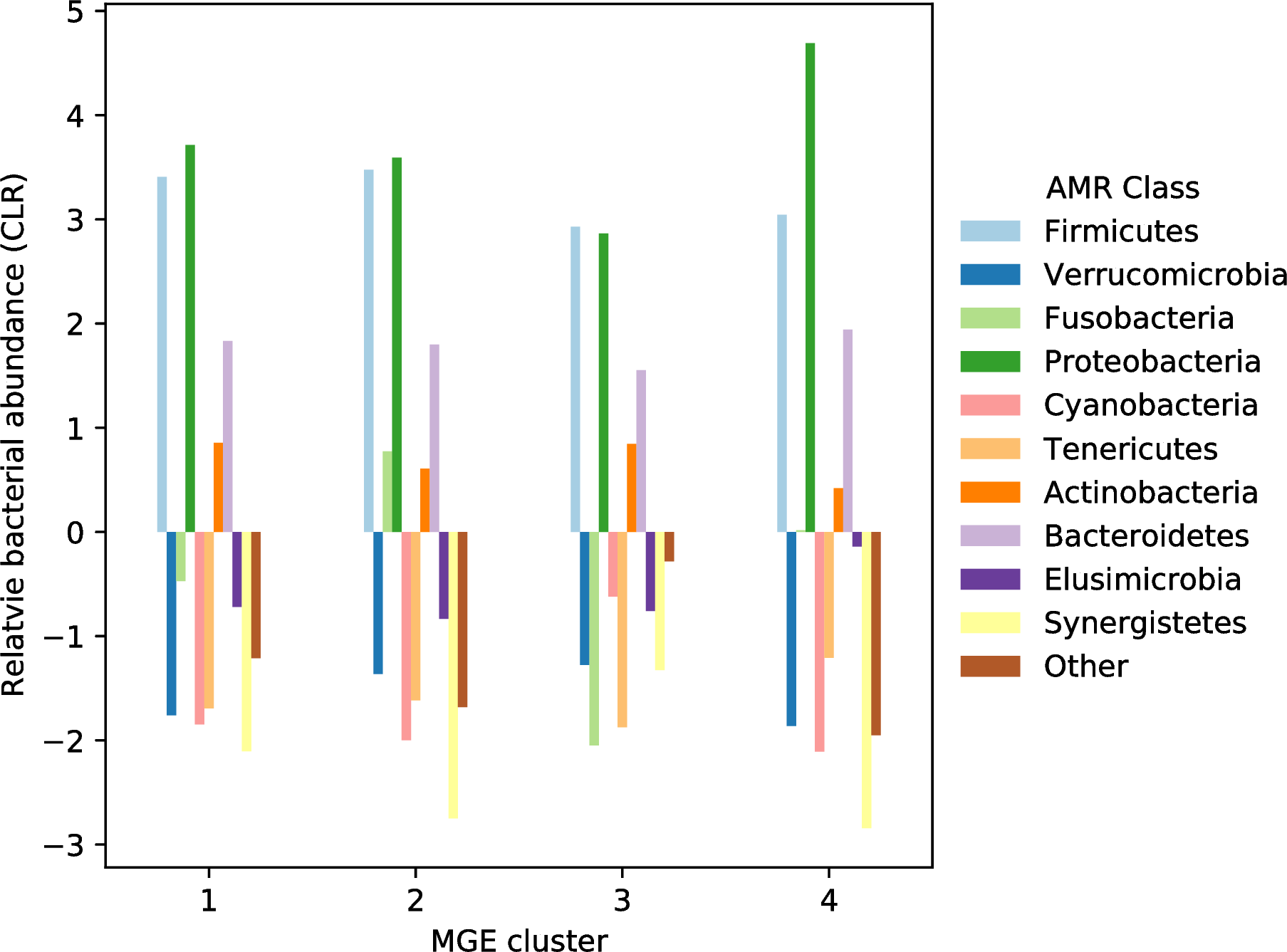

Supplement: S6 Fig — Bacterial abundance was estimated from the number of fragments mapping to 16S. Phyla with a relative frequency lower than 0.05% of all mapped was combined into the other category. Abundances are CLR transformed. Samples in cluster 1 and 2 has higher content of Firmicutes than cluster 3 and 4; cluster 2 has higher content of Fusobacteria and cluster 4 has higher content of Proteobacteria. (TIF) [file pone.0293169.s006.tif]

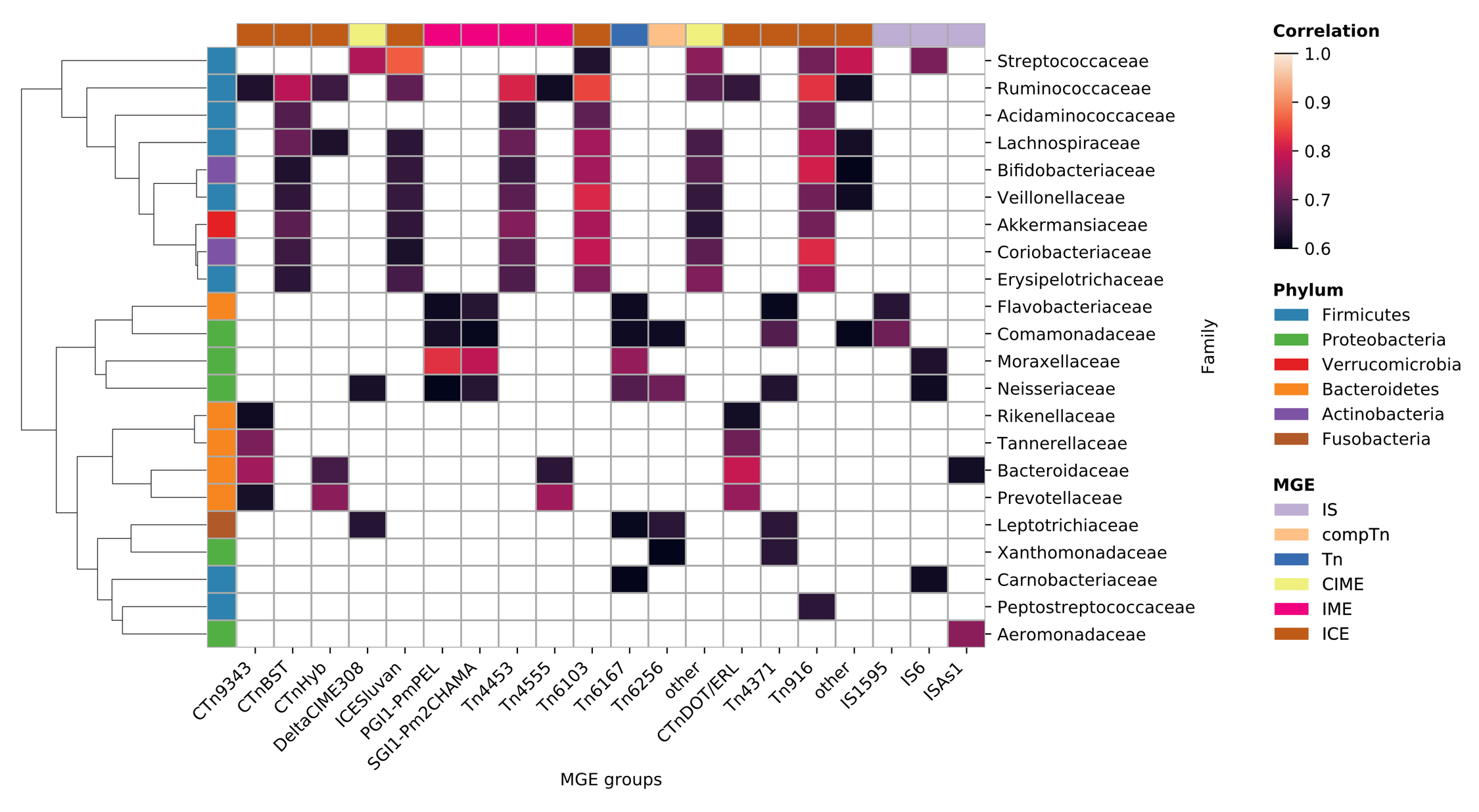

Supplement: S7 Fig — MGEs are colored on the type and taxonomic families are colored according to their phyla. Correlations was calculated on the relative abundance of homology reduced MGEs and only significant correlations was included. The rows were clustered using average linkage to display MGEs spanning multiple phyla. (TIF) [file pone.0293169.s007.tif]

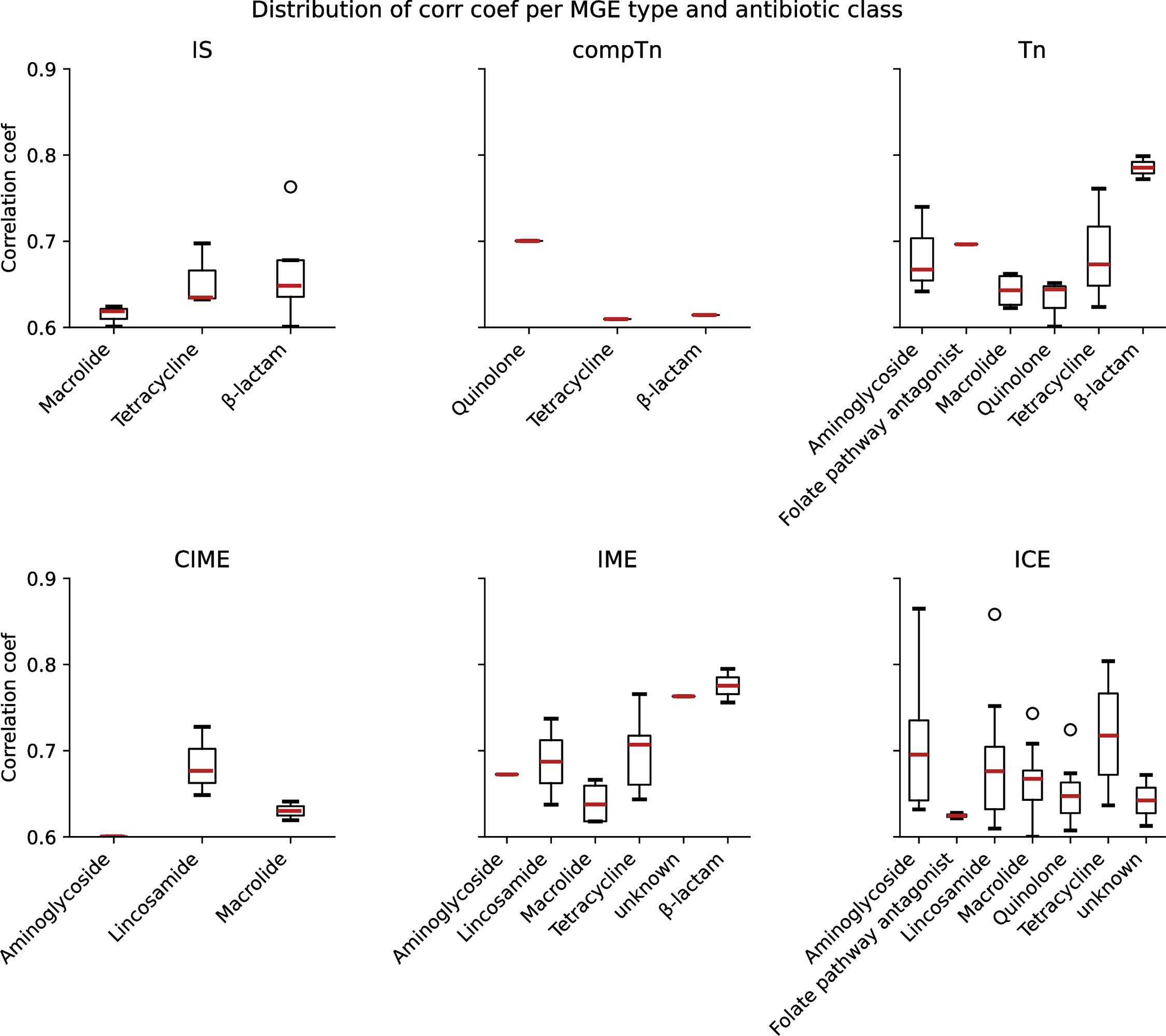

Supplement: S8 Fig — (TIF) [file pone.0293169.s008.tif]

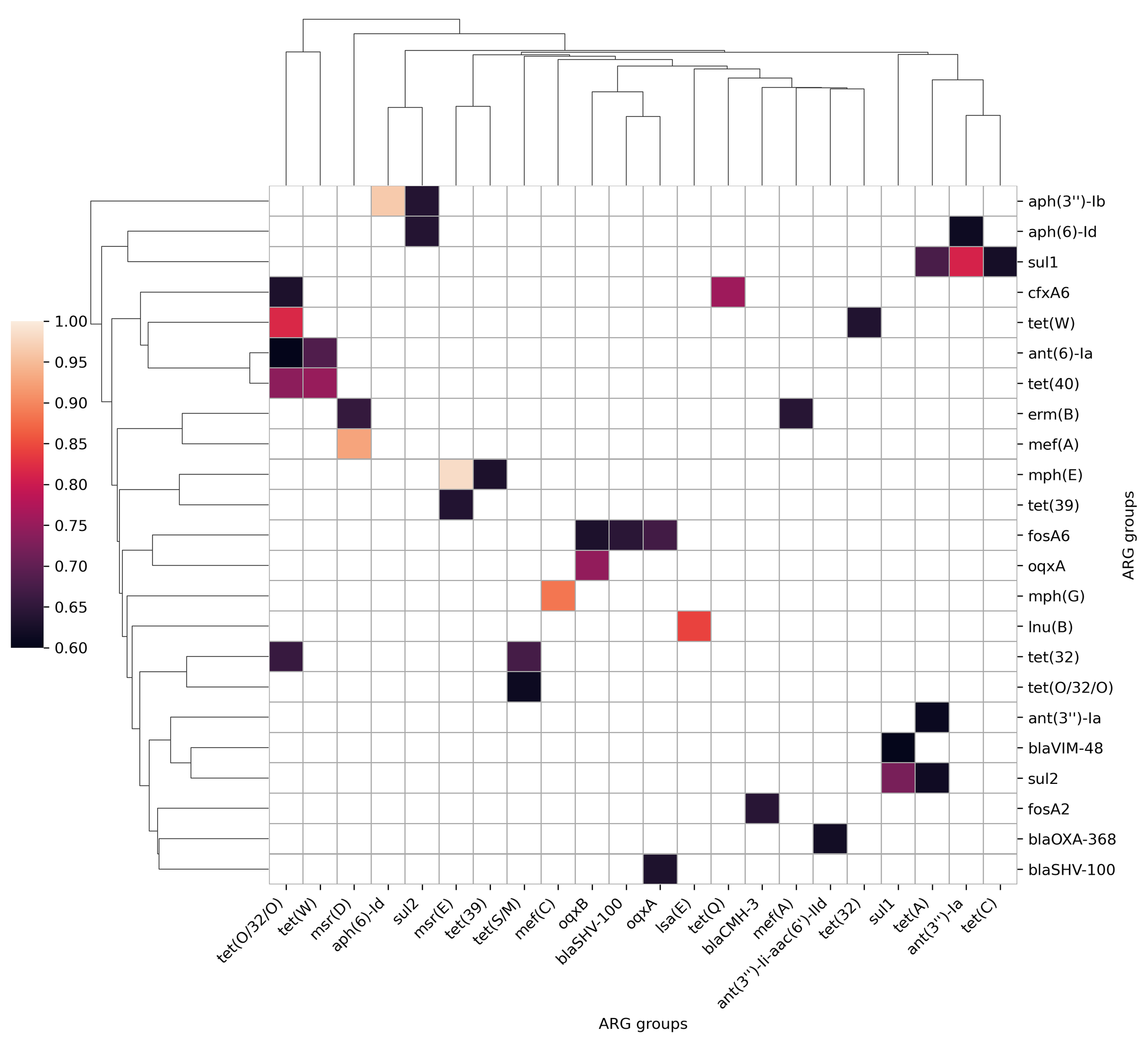

Supplement: S9 Fig — Correlations was calculated on the relative abundance of homology reduced genes and only significant correlations was included. ARG groups were clustered using average linkage. (TIF) [file pone.0293169.s009.tif]
