# Supplementary material for: Importance of mobile genetic elements for dissemination of antimicrobial resistance in metagenomic sewage samples across the world
Source: PLoS One. 2023 Oct 19;18(10):e0293169. doi: 10.1371/journal.pone.0293169 (PMC10586675; doi:10.1371/journal.pone.0293169)
Supplement: S1 Table — (DOCX) [file pone.0293169.s010.docx]

| **Database** | **KMA parameters** |
| --- | --- |
| **ResFinder** | mem_mode; 1t1; cge |
| **mgeDb** | mem_mode; 1t1; apm f |
| **Silva** | mem_mode; 1t1; apm f |
