# Supplementary material for: Importance of mobile genetic elements for dissemination of antimicrobial resistance in metagenomic sewage samples across the world
Source: PLoS One. 2023 Oct 19;18(10):e0293169. doi: 10.1371/journal.pone.0293169 (PMC10586675; doi:10.1371/journal.pone.0293169)
Supplement: S2 Table — Shannon information was calculated from these observations and quantifies the randomness of the distribution where 0 is not random and 1 is evenly distributed. (DOCX) [file pone.0293169.s011.docx]

| **Type of genetic element** | **Cluster** | **Samples from frigid & temperate zone** | **Samples from subtropic & tropic** | **Shannon Information [bit]** |
| --- | --- | --- | --- | --- |
| ***MGEs*** | 1 | 12 | 219 | 0.295 |
|  | 2 | 243 | 15 | 0.320 |
|  | 3 | 9 | 53 | 0.598 |
|  | 4 | 39 | 75 | 0.927 |
| ***ARGs*** | 1 | 3 | 184 | 0.119 |
|  | 2 | 266 | 56 | 0.667 |
|  | 3 | 34 | 68 | 0.918 |
|  | 4 | 0 | 54 | 0 |
