# Supplementary material for: Importance of mobile genetic elements for dissemination of antimicrobial resistance in metagenomic sewage samples across the world
Source: PLoS One. 2023 Oct 19;18(10):e0293169. doi: 10.1371/journal.pone.0293169 (PMC10586675; doi:10.1371/journal.pone.0293169)
Supplement: S3 Table — MGE groups are named after the representative MGE or MGE family. (DOCX) [file pone.0293169.s012.docx]

| **MGE group** | **MGE type** | | **Correlated ARGs** |
| --- | --- | --- | --- |
| Tn*916* | ICE | ant(6)-Ia, erm(B), tet(40), tet(O/32/O), tet(W) | |
| Tn*6167* | Unit transposon | blaOXA-280, mph(E), msr(E), tet(39) | |
| Tn*6171* | Unit transposon | blaOXA-280, mph(E), msr(E), tet(39) | |
| SGI1-Pm2CHAMA | IME | blaOXA-280, mph(E), msr(E), tet(39) | |
| PGI1-PmPEL | IME | blaOXA-280, mph(E), msr(E), tet(39) | |
| Tn*6103* | ICE | ant(6)-Ia, erm(B), tet(O/32/O), tet(W) | |
| IS*701* family | Insertion sequence | blaOXA-280, mph(E), msr(E), tet(39) | |
| Tn*5801* | ICE | erm(B), mef(A), msr(D) | |
| ICEKkKWG1 | ICE | aph(3'')-Ib, aph(6)-Id, sul2 | |
| Tn*4453* | IME | ant(6)-Ia, erm(B), tet(W) | |
